# Knowledge Regarding Hypoglycemia and Its Management Among Patients With Insulin-Requiring Diabetes Mellitus in Al-Ahsa, Saudi Arabia

**DOI:** 10.7759/cureus.47257

**Published:** 2023-10-18

**Authors:** Hessah Al Hussaini, Ali Alismael, Mohammed Alquraini, Thamer Alhabdan, Hassan Alramadan, Jumanah Alqattan, Sayed Ali, Bashaeer Aljalal, Mohammed Almulhim, Muthana Al Sahlawi

**Affiliations:** 1 Internal Medicine, College of Medicine, King Faisal University, Al-Ahsa, SAU; 2 Medical School, College of Medicine, King Faisal University, Al-Ahsa, SAU; 3 Family & Community Medicine, College of Medicine, King Faisal University, Al-Ahsa, SAU

**Keywords:** insulin-dependent diabetes mellitus, management, knowledge, hypoglycemia, insulin, diabetes mellitus

## Abstract

Background: Hypoglycemia has a negative influence on patients with diabetes mellitus (DM) using insulin, and a lack of knowledge about hypoglycemia is one of the main causes of hypoglycemia. We aim to assess the level of knowledge about hypoglycemia and its management among insulin-requiring DM patients in Al-Ahsa, Saudi Arabia.

Methods: A cross-sectional study was conducted on patients with insulin-requiring DM in Al-Ahsa, Saudi Arabia, from November 2021 to March 2022. A questionnaire was filled out by the participants to assess their level of knowledge about hypoglycemia, which was categorized as good, fair, or poor if participants scored >7, 6-7, or <6, respectively.

Results: A total of 238 participants were recruited. Among participants, 55% were males, mainly in the age group of 40-65 years, and had higher education degrees. Doctors were the main source of information among participants. Most patients had no chronic illnesses other than DM or DM-related complications, although hypertension was reported in 37% of patients. The majority of participants had a good level of knowledge regarding hypoglycemia, and the main factors that increased it were getting information from doctors, social media, or a booklet or pamphlet (p-value <0.05).

Conclusion: Our participants generally had a good level of knowledge about hypoglycemia, and doctors delivering information about hypoglycemia contributed significantly to this knowledge level.

## Introduction

Diabetes mellitus (DM) is among the world's most prevalent chronic illnesses, with approximately 537 million adults and 1.52 million children and adolescents having diabetes, and this number is anticipated to rise in the coming years [1-2]. In 2021, the number of diabetic patients in the Kingdom of Saudi Arabia was 289,000 (0-19 years) and 4.3 million (20-79 years), which is among the highest nations in the world and the top five countries in the Middle East and North Africa [1].

Diabetes mellitus can lead to several acute consequences; one of them is hypoglycemia due to insulin use, which is a critical medical emergency seen in people with either type 1 diabetes (T1D) or type 2 diabetes (T2D) [3]. Hypoglycemia is two to three times more common in T1D than it is in T2D. However, as T2D is more prevalent than T1D, patients with T2D experience the majority of hypoglycemia episodes [4]. According to the American Diabetes Association, hypoglycemia is considered when the blood glucose level is lower than 3.9 mmol/L (70 mg/dl), and the severity of hypoglycemia is determined based on the patient's need for assistance in managing hypoglycemia [5]. The frequency of hypoglycemia among diabetic patients is unknown, and it varies from patient to patient depending on their health status. However, diabetic patients experience 23 episodes of mild or moderate hypoglycemia per year and at least one severe hypoglycemia episode per year [6]. Patients' failure to acknowledge hypoglycemia symptoms and a lack of understanding about how to handle them correctly are common causes of hypoglycemia episodes, which can lead to multi-organ complications and increase morbidity and mortality, especially with severe hypoglycemia (hypoglycemia associated with loss of consciousness) [3,7]. Furthermore, hypoglycemia is infrequently reported to healthcare providers by 43%-53% of patients with T2D [8]. Since insulin is the major cause of hypoglycemia in diabetic patients, our study aims to assess the knowledge about hypoglycemia and its management among insulin-dependent diabetes mellitus patients in Al-Ahsa, Saudi Arabia.

## Materials and methods

This cross-sectional study was conducted from November 2021 to March 2022 among diabetic patients who live in Al-Ahsa, Saudi Arabia, in all age groups, with any type of DM, both genders, all nationalities, and who used insulin as a part of their DM management. We aimed to assess their knowledge about hypoglycemia and its management. Our questionnaire was reviewed for face and content validity by 10 experts in the fields of internal medicine, diabetes, and endocrinology and was also tested for reliability by 30 participants (pilot). The questionnaire was distributed online via social media and filled out by the participants, who were either patients themselves or the patient's caregivers. Caregivers are those who are responsible for DM management in enrolled patients who cannot take care of their DM management alone (for example, patients under 18 years of age).

The questionnaire was divided into three sections. The first section included questions about the demographic characteristics of participants (six questions), the second section included questions about DM history (four questions), and the third section included questions about knowledge of hypoglycemia (13 questions). After the data were extracted, they were revised, coded, and fed to IBM Statistical Package for the Social Sciences (SPSS) software version 26 (IBM Corp., Armonk, NY). The categorical variables were summarized using frequency and percentage. Statistical significance was considered at a p-value<0.05 and a confidence interval (CI) of 95%. Most of the questions had only one correct answer (nine out of 13 questions). However, some of the questions had multiple correct answers (four out of 13 questions), so participants who answered at least two questions correctly were scored as correct. A score of one was given for each question answered correctly, and a score of 0 was given for the wrong answer. The summation of scores was computed (the total score was 13). Knowledge scores were categorized into good (if score >7, i.e., >60%), fair (if score 6-7), and poor (if score <6, i.e., <50%).

We recruited 238 participants based on a sample size calculation. A sample size of 223 patients was considered a representative sample of DM patients in Saudi Arabia. It was calculated based on the following formula: n=p(1-p)z2/e2, where p=prevalence of DM patients in Saudi Arabia, which is 17.7% according to the International Diabetes Federation (IDF) in the year 2021 [2], z=1.96 for a confidence level (α) of 95%, and e=margin of error of 0.05.

The ethical approval was obtained from the Research Ethical Committee at King Faisal University, Al-Ahsa, Saudi Arabia (approval number: KFU-REC-2021-NOV-EA000154). Informed consent was obtained from all participants before their participation, and they had the right to withdraw from the study. Also, they were informed that their participation was confidential and that their information would be used only for research purposes.

## Results

We collected 238 participants in our study. The participants were predominantly males (n=132, 55.5%), with a high percentage being between the age range of 40 and 65 years old (n=109, 45.8%). Regarding marital status, most of the participants were married (n=150, 63%). Considering educational level, 142 participants (59.7%) had higher education degrees (any degree after a high school degree). More than half of patients don’t have chronic illnesses other than DM (n=139, 58.4%). If they did, hypertension was the most common chronic illness in our patients (n=88, 37%) (Table 1).

**Table 1:** Participants' characteristics ^a^ The number of responses can be more than the overall number of participants because they can select more than one answer. DM: diabetes mellitus

Characteristic	N	%
Patient age		
Less than 5 years	3	1.3%
5 – 14 years	22	9.2%
15 – 24 years	39	16.4%
25 – 39 years	38	16%
40 – 65 years	109	45.8%
More than 65 years	27	11.3%
Gender		
Male	132	55.5%
Female	106	44.5%
Marital status		
Single	71	29.8%
Married	150	63%
Widowed	17	7.1%
Educational level		
Not educated	10	4.2%
Primary school	21	8.8%
Intermediate school	21	8.8%
High school	44	18.5%
Higher education	142	59.7%
Chronic diseases other than DM ^a^		
No chronic diseases	139	58.4%
Hypertension	88	37%
Renal impairment	13	5.5%
Ischemic heart disease	15	6.3%
Stroke	5	2.1%

Among the enrolled patients, 122 patients had T2D (51.3%), 111 of them had T1D (46.6%), and five patients were unaware of their DM type (2.1%). Among all patients, 121 were using insulin as well as other DM medications to manage DM (50.8%), compared to 117 who were using only insulin (49.2%) for their DM management. In the study group, 161 patients reported no DM complications (67.6%). However, DM retinopathy (48 patients, 20.2%) and neuropathy (36 patients, 15.1%) were the most reported complications among those with DM complications (Table 2).

**Table 2:** The participants' DM history ^a ^The number of responses can be more than the overall number of participants because they can select more than one answer. DM: diabetes mellitus

Characteristic	N	%
Type of DM		
Type 1	111	46.6%
Type 2	122	51.3%
Unknown	5	2.1%
Medications		
Only insulin	117	49.2%
Insulin and other medications	121	50.8%
Complications of DM ^a^		
No complications	161	67.6%
DM retinopathy	48	20.2%
DM nephropathy	15	6.3%
Stroke	7	2.9%
Ischemic heart disease	10	4.2%
Diabetic foot ulcer	12	5%
DM neuropathy	36	15.1%

Most of the participants reported that their main source of information about hypoglycemia was from doctors (73.5%). Others reported that their sources were relatives or friends, social media, booklets or pamphlets, or websites (34.9%, 25.6%, 16%, and 14.7%, respectively) (Table 3).

**Table 3:** Sources of information about hypoglycemia ^a^ The number of responses can be more than the overall number of participants because they can select more than one answer.

Source of information ^a^	N		%
Doctor	175	73.5%	
Relative or friend	83	34.9%	
Website	35	14.7%	
Social media	61	25.6%	
Booklet/Pamphlet	38	16%	

Most of the questions had only one correct answer. However, some of the questions had multiple correct answers, so participants who answered at least two questions correctly were scored as correct. Most participants answered each question correctly. Among all 13 questions (Table 4), we noticed five questions were answered incorrectly by more than 50% (question numbers 3, 5, 10, 12, and 13), which indicates less information about hypoglycemia.

**Table 4:** Correct answers to the knowledge assessment about hypoglycemia questions

Questions	N	%
1. What is the level of blood sugar that defines hypoglycemia?	166	69.7%
2. What is the level of blood sugar that implies severe hypoglycemia?	168	70.6%
3. Knowledge about glucagon	83	34.9%
4. What are the symptoms of hypoglycemia?	210	88.2%
5. Is it possible to have hypoglycemia without symptoms?	111	46.6%
6. What are the causes of hypoglycemia?	159	66.8%
7. What are the major complications of hypoglycemia?	105	44.1%
8. Hypoglycemia attacks are most commonly observed in patients with...	121	50.8%
9. How can hypoglycemia be managed in a fully awake patient at home?	191	80.3%
10. How can severe hypoglycemia be managed in a drowsy patient at home?	103	43.3%
11. When does blood glucose need to be rechecked after hypoglycemia management?	124	52.1%
12. When should diabetic patients break fasting?	104	43.7%
13. When should diabetic patients avoid driving?	100	42%

The total number of questions to assess the knowledge of hypoglycemia was 13. Those who answered eight or more correctly were considered to have a good level of knowledge, and they represented 87% of all participants. Those who answered six to seven questions correctly are considered to have a fair level of knowledge (6.7%). Those who answered five or fewer questions correctly were considered to have a poor level of knowledge (6.3%) (Table 5).

**Table 5:** Participants' level of knowledge about hyperglycemia

Level of knowledge about hypoglycemia	N	%
Poor	15	6.3%
Fair	16	6.7%
Good	207	87%

The level of knowledge was higher among female participants (92% had good knowledge vs. 82.6% for male participants), those with higher educational level (89.4% had good knowledge vs. 70% for participants who were not educated), singles (88.7% had good knowledge vs. 86% for married participants), and the participants in the age range between 25 and 39 years (97.4% had good knowledge vs. 66.7% among those aged less than five years), although all had statistically insignificant relation (p-value>0.05 for all). In regards to chronic illnesses, 94.3% of patients with hypertension had a good level of knowledge, in comparison to 82.1% of patients without hypertension, and this was statistically significant (p-value=0.016). In comparison to other comorbidities (renal impairment, ischemic heart disease, and stroke), there was no statistical significance in the level of knowledge about hypoglycemia (p-value>0.05) (Table 6).

**Table 6:** Relationship between participant demographics and knowledge about hyperglycemia * p< 0.05 (significant)

Characteristic	Level of knowledge						p-value
	Good		Fair		Poor		
	N	%	N	%	N	%	
Age							
Less than 5 years	2	66.7%	1	33.3%	0	0	0.193
5 – 14 years	21	95.5%	0	0	1	4.5%	
15 – 24 years	33	84.6%	4	10.3%	2	5.1%	
25 – 39 years	37	97.4%	0	0	1	2.6%	
40 – 65 years	93	85.3%	7	6.4%	9	8.3%	
More than 65 years	21	77.8%	4	14.8%	2	7.4%	
Gender							
Male	109	82.6%	12	9,1%	11	8.3%	0.079
Female	98	92%	4	3.8%	4	3.8%	
Marital status							
Single	63	88.7%	3	4.2%	5	7%	0.628
Married	129	86%	11	7.3%	10	6.7%	
Widow	15	88.2%	2	11.8%	0	0	
Educational level							
Not educated	7	70%	3	30%	0	0	0.117
Primary school	19	90.5%	0	0	2	9.5%	
Intermediate school	17	81%	2	9.5%	2	9.5%	
High school	37	84.1%	3	6.8%	4	9.1%	
Higher education	127	89.4%	8	5.6%	7	4.9%	

The source of information about hypoglycemia among participants was tested to assess the level of knowledge about hypoglycemia. Doctors, social media, and booklets or pamphlets as sources of information were associated with better knowledge among our participants, and they were statistically significant (92.4%, 96.6%, and 100%, respectively, with a p-value<0.05). There was also a better level of knowledge among participants who have relatives or friends or used websites as sources of information about hypoglycemia, but they were statistically insignificant (Table 7).

**Table 7:** Relationship between sources of information and knowledge regarding hyperglycemia * p<0.05 (significant)

Source of information	Level of knowledge						p-value
	Good		Fair		Poor		
	N	%	N	%	N	%	
Doctor							
Yes	158	92.4%	8	4.7%	5	2.9%	0.000*
No	44	71%	8	12.9%	10	16.1%	
Relative or friend							
Yes	72	88.9%	5	6.2%	4	4.9%	0.745
No	130	85.5%	11	7.2%	11	7.2%	
Website							
Yes	30	88.2%	3	8.8%	1	2.9%	0.610
No	172	86.4%	13	6.5%	14	7%	
Social media							
Yes	56	96.6%	0	0%	2	3.4%	0.027*
No	146	83.4%	16	9.1%	13	7.4%	
Booklet/Pamphlet							
Yes	38	100%	0	0%	0	0%	0.031*
No	164	84.1%	16	8.2%	15	7.7%	

In regards to DM type and medications used, there was no difference in the level of knowledge about hypoglycemia between T1D and T2D or among patients using insulin with or without other medications (p-value>0.05) (Table 8).

**Table 8:** Relationship between DM history and knowledge about hyperglycemia *p< 0.05 (significant) DM: diabetes mellitus

Characteristic	Level of knowledge						p-value
	Good		Fair		Poor		
	N	%	N	%	N	%	
Type of DM							
Type 1	96	86.5%	8	7.2%	7	6.3%	0.979
Type 2	106	86.9%	8	6.6%	8	6.6%	
Medications							
Only insulin	106	90.6%	5	4.3%	6	5.1%	0.234
Insulin and other DM medications	101	83.5%	11	9.1%	9	7.4%	

## Discussion

Due to its rising prevalence over the past few decades, diabetes has become one of the greatest public health concerns due to its short- and long-term complications [9]. One of the most common problems with managing diabetes is hypoglycemia [10]. The lack of awareness about hypoglycemia is among the significant factors that increase the incidence of hypoglycemia [11]. The current study aimed to assess the knowledge about hypoglycemia and its management among insulin-requiring DM patients. The majority of the study's participants (87%) demonstrated good knowledge of hypoglycemia. Similar findings have been identified in a study carried out by Sharma et al., who found that 64.4% of diabetic patients had good knowledge of hypoglycemia [12]. However, our study findings contradict a previous study that was conducted in Jeddah and found that 92.2% of participants had poor knowledge of hypoglycemia [3]. This difference in knowledge can be attributed to many factors. One factor is the scoring system, which categorizes participants' knowledge as good, fair, or poor. The latter study had a thorough assessment with a total score of 42, where an individual was considered to have good knowledge if the provided score was higher than 31.5 (>75%), which indicates a higher expectation from the patient about hypoglycemia knowledge. In our study, we consider a level of knowledge of >60% (i.e., seven out of 13) to be a good level of knowledge about hypoglycemia. Another possible factor is the educational level because most of our participants have a higher education (59.7%) compared to 8% in Jeddah, so the educational level can be beneficial to clearly understanding the instructions from their physicians or a health care professional. Although insignificant, we found that female participants (92%) and participants using only insulin (90.6%) had a better level of knowledge in comparison to male participants (82.6%) and participants using medications for DM other than insulin (83.5%), with a p-value of 0.079. We didn't find any difference in the level of knowledge about hypoglycemia between DM types, and this is different from a study conducted in Jeddah that showed patients with T1D have better knowledge [3]. Our results showed that the question on symptoms of hypoglycemia was the most correctly answered (88.2%), and dizziness was the most common symptom of hypoglycemia (71.4%), followed by sweating (67.2%) and shaking or tremors (66.4%). In comparison, another study done in Riyadh showed that dizziness is the most common symptom of hypoglycemia (74.6%), followed by excessive hunger (73.1%) and sweating (71.8%) [13]. Nevertheless, another study done in Al-Ahsa showed the most common symptoms of hypoglycemia were tremors (69.7%), sweating (64%), palpitation (61.8%), and drowsiness (56.2%) [14]. However, high percentages of dizziness and sweating in these studies support the fact that they are frequently encountered symptoms of hypoglycemia. In this current study, 73.5% of the participants received their information from a doctor, which is a similar result noticed in Kalantzi’s study in Greece, which reported that the majority of participants rely on their physicians as their source of information [15]. Participants who reported doctors as their source of information on hypoglycemia had a good level of knowledge (92.4%), while just 2.9% had a poor level of knowledge. On the other hand, among participants who did not get their information from doctors, 71% had a good level of knowledge, while 16.1% had a poor level of knowledge. There is no previous finding in the literature that specifically discusses the association between the level of knowledge and the source of information. These findings suggest that getting information from a doctor is related to a higher level of knowledge about hypoglycemia.

Even though the level of awareness about hypoglycemia is extremely excellent in our study, a group of participants had a misunderstanding of some crucial features of hypoglycemia, such as its complications and some elements of management. Health education on various elements of hypoglycemia at every visit by the attending physicians and nurses is important to raise awareness and avoid hypoglycemia. Also, increasing the effort using different methods of information dissemination, such as social media and social networking platforms, must be taken into consideration.

The strengths of this study include the valuable insight it provides into the level of knowledge about hypoglycemia and its management among insulin-requiring diabetic patients in Al Ahsa, Saudi Arabia. Additionally, the use of a structured questionnaire allowed for standardized data collection and analysis, and the identification of areas where patients had good knowledge about hypoglycemia and areas where patients had less knowledge can help healthcare providers target their education efforts. However, there are also some limitations to this study. First, the study was conducted in only one city in Saudi Arabia, which limits the generalizability of the findings to other regions or countries. Second, the study focused only on insulin-requiring diabetic patients, so the results may not apply to all diabetic patients. Third, we didn't include the type of insulin used, as hypoglycemia risk is variable depending on insulin type. Finally, the study relied on self-reported data, which may be subject to bias and may not reflect the actual knowledge and behavior of the participants. Further studies in multiple regions with larger sample sizes and further detailed information assessed can be more generalizable.

## Conclusions

We conclude that our participants have excellent knowledge about hypoglycemia, and this can be attributed to the participants' high level of education and reliance on doctors, social media, and booklets or pamphlets as the main sources of information about hypoglycemia. Nevertheless, some information about hypoglycemia complications and management is deficient among our participants. This issue should be addressed and improved in our daily practice.
